# A modular cGAN classification framework: Application to colorectal tumor detection

**DOI:** 10.1038/s41598-019-55257-w

**Published:** 2019-12-12

**Authors:** Thomas E. Tavolara, M. Khalid Khan Niazi, Vidya Arole, Wei Chen, Wendy Frankel, Metin N. Gurcan

**Affiliations:** 1 Center for Biomedical Informatics, Wake Forest School of Medicine, Winston-Salem, USA; 2 Department of Pathology, The Ohio State University, Columbus, USA

**Keywords:** Rectal cancer, Colon cancer, Colon cancer, Colon cancer, Rectal cancer

## Abstract

Automatic identification of tissue structures in the analysis of digital tissue biopsies remains an ongoing problem in digital pathology. Common barriers include lack of reliable ground truth due to inter- and intra- reader variability, class imbalances, and inflexibility of discriminative models. To overcome these barriers, we are developing a framework that benefits from a reliable immunohistochemistry ground truth during labeling, overcomes class imbalances through single task learning, and accommodates any number of classes through a minimally supervised, modular model-per-class paradigm. This study explores an initial application of this framework, based on conditional generative adversarial networks, to automatically identify tumor from non-tumor regions in colorectal H&E slides. The average precision, sensitivity, and F1 score during validation was 95.13 ± 4.44%, 93.05 ± 3.46%, and 94.02 ± 3.23% and for an external test dataset was 98.75 ± 2.43%, 88.53 ± 5.39%, and 93.31 ± 3.07%, respectively. With accurate identification of tumor regions, we plan to further develop our framework to establish a tumor front, from which tumor buds can be detected in a restricted region. This model will be integrated into a larger system which will quantitatively determine the prognostic significance of tumor budding.

## Introduction

One of the fundamental problems in the analysis of digital tissue biopsies is the identification of anatomical structures^1–3^. It is often a key first step to the automated analysis of many diseases, most prevalently cancer. As a result, one of the most recurrent manifestations of this problem is identification of malignant or metastatic tissue^4–6^. This is important, as the extent to which malignant tissue has invaded neighboring tissues or metastasized is critical for long term prognosis as well as treatment options. Further, it is important for the establishment of novel prognostic factors – for example, in the substaging of digestive tract cancers by depth of connective tissue invasion^7^ or to determine the ratio of immunohistochemistry (IHC) positive cells in tissue substructures^4,8^. However, the development of these kinds of automated systems (especially deep learning) is hindered by several factors.

The first of these factors is the inherent inter- and intra- reader variability in the interpretation of tissue biopsies. The decision by a single pathologist may differ from that of another pathologist and may even differ day to day from the same pathologist^2,9,10^. This is especially detrimental to patients but also to the development of those systems that seek to resolve the very same problem, as it is difficult to generate a ground truth. IHC staining, due to its ability to visualize specific disease-associated proteins, can mitigate these problems but remains relatively more expensive and restricted in terms of availability compared to standard H&E^11^. Current solutions to this ground truth generation problem include pathologist consensus, but even then, there will always be disagreement^10,12^.

Class imbalance is a second hindrance. It is the lack of training data available for one or more classes relative to other classes in the training of classifiers. Such imbalances cause a model to bias towards classifying unknown entities as classes with a larger number of samples as a result of the optimization process during training^13^. This is especially a problem in the domain of automated medical image analysis and computational pathology alike^14^. Typical solutions include resampling datasets using bagging or boosting, but this often results in drastically smaller datasets and thus is undesirable. Other methods include generating synthetic samples^15^, penalizing the classification models^16^, however, their generalization to unseen data is at least questionable.

Finally, a third problem in the development of automated tissue analysis systems is the inherent fixed number of classes in discriminative models. Development of a new classification method in digital pathology involves changing the number of classes by grouping certain anatomical structures into a single bin or by splitting a class into two or more, coincidentally often due to class imbalances. Though there is no theoretical problem in doing this, it is often impractical, as one must retrain the entire model based on the number of classes being considered.

We propose a framework that seeks to resolve these problems using a modular, minimally-supervised, model-per-class framework applied to the identification of tumor and non-tumor regions in digital tissue biopsies of colorectal cancer (CRC). As tumor regions are easily identified using pan-cytokeratin staining^17^, our ground truth is generated from manual registration of adjacent pan-cytokeratin and H&E slides. Thus, the system benefits from accurately segmented pan-cytokeratin and H&E slides during the training and validation stages but relies solely on H&E for testing and deployment, thereby resolving inter- and intra- reader variability.

The proposed framework combines two independently trained conditional generative adversarial networks (cGANs) to identify tumor and non-tumor regions. cGANs and its variants have been utilized in numerous digital pathology problems^2^, including, but not limited to color normalization^18^, generation of artificial high-resolution breast cancer biopsies^15^, and virtual staining of hyperspectral images^19^. However, its ability to classify different anatomical structures from H&E images is hardly explored. This is an innovative application of generative models to classify tissue structures.

These two cGANs are trained to reconstruct 256 × 256 pixel images extracted from respective tumor and non-tumor H&E stained CRC biopsies. Theoretically, a cGAN trained on tumor images learns a latent space to reconstruct tumor regions. Similarly, the other cGAN learns a different latent space to reconstruct non-tumor regions. After training, as a test image is presented to the system, both cGANs attempt to reconstruct the test image. The image is classified based on the reconstruction that is most similar to the test image. Using these strategies, class imbalance is effectively a non-problem, as the tumor and non-tumor models are separate. Further, given the generative nature of cGANs, the retraining and fixed class problems of typical discriminative models is effectively resolved, as addition or removal of classes simply involves training of a single model per class without modifying other models.

We utilize Inception v3^20^ trained via transfer learning as a baseline comparison, as it has been shown to effectively identify various histopathological anatomies including tumor bulk in several previous studies^2,4,7,21–26^. Briefly, the majority of these methods sample tumor and non-tumor regions at some magnification and tile size then train a convolutional neural network to discriminate between the two classes either through transfer learning, fine-tuning, or from scratch.

Both our method and Inception v3 are assessed with 4-fold cross-validation using an initial dataset of 24 adjacent pan-cytokeratin and H&E slides. Finally, the proposed method is evaluated on an external test dataset of 91 slides and evaluated against a pathologist’s ground truth in terms of precision, sensitivity, and F1 score.

## Results

### Optimization of tile size and magnification

An initial pilot experiment was trained with 64 × 64 tiles at 40x magnification rather than 256 × 256 tiles. This initial decision for a 64 × 64 tile size was motivated by a the same choice made by a previous study applying Inception to segment pancreatic neuroendocrine tumor from stroma from Ki67 stained slides^7^. The dataset was derived from our 24 slide training/validation dataset using leave-one-slide-out. Tumor and non-tumor tiles were extracted from each slide. Due to the low accuracy of pilot models, we decided to run an brute force optimization of tile size and magnification using transfer learning (Inception v3) as described in^4^. Each Inception v3 model was trained using a leave-one-out method with the parameters described in the Methods section. Six varying tile size and magnification combinations were used – 64 × 64 at 10x, 64 × 64 and 128 × 128 at 20x and 40x, and 256 × 256 at 40x. Lower magnifications and larger tile sizes were not considered, as the number of resulting tiles was too small (<1000 per class). Supplementary Fig. S1 shows the results of this tile size and magnification optimization experiment.

Based on a sum of the averaged training and validation accuracies across each leave-one-out training session and the resulting averaged testing accuracies, 256 × 256 at 40x was determined to be the optimal tile size and magnification, with 128 × 128 at 20x coming in a close second place. It is worth mentioning that these two tile sizes and zoom combinations refer to the same *physical* tile size, i.e. they occupy the same physical area when superimposed under a microscope. These results imply that a specific tile size and magnification are required for deep learning models. Subsequent experimentation and the results presented in the remainder of this section are based off this optimization, i.e. utilizing a dataset sampled for 256 × 256 pixel tiles at 40x magnification. In the context of histopathological image analysis, this tile size matches those of our previous work^27,28^.

### Inception as a baseline comparison

To determine a baseline comparison, Inception v3 was trained and validated on each cross-validation dataset (as described in Methods). Results of Inception are shown in Table 1. In general, Inception performed exceptionally well, scoring more than 95% on precision, sensitivity, and F1 for the majority of configurations. Remarkably, the lowest score on these particular measures was 82% (see Discussion for details). There was no considerable variability among these measures across each set, with the exception of the precision on the validation set, which varied between 82% to 97% with an average of 90.75%.

**Table 1:** Training and validation precision, sensitivity, and average F1 and standard deviations across each fold trained on Inception v3.

Training			Validation		
Precision	Sensitivity	F1	Precision	Sensitivity	F1
95.05 ± 1.11	98.91 ± 0.12	96.94 ± 0.52	90.69 ± 5.77	96.77 ± 3.17	93.45 ± 2.59

### cGAN classification by minimum MSE

Our model trains two cGANs – one on tumor tiles and one on non-tumor tiles. As an unlabeled tile is presented to the network, the *edge condition* is applied, and the tile is passed through both tumor and non-tumor generators. The generator that produces a reconstruction with the smallest MSE is declared the ‘winner’ and the tile is labeled as such. Additional image quality metrics were tested, such as SSIM^29^ and peak-signal-to-noise-ratio, but these were slower and generally produced the similar classification results. Table 2 summarizes the results of applying the proposed model to a 4-fold cross validation (6 slides per fold) for Canny edge conditioning. See the Methods section for details on this cross-validation approach.

**Table 2:** Training and validation precision, sensitivity, and average F1 and standard deviations across each fold trained using cGANs with two Canny edge conditions.

Condition	Training			Validation		
	Precision	Sensitivity	F1	Precision	Sensitivity	F1
sigma_2	99.24 ± 0.32	97.20 ± 1.73	98.21 ± 0.92	94.55 ± 5.04	93.20 ± 1.85	93.80 ± 2.97
sigma_5	99.30 ± 0.51	97.54 ± 1.13	98.40 ± 0.39	95.13 ± 4.44	93.05 ± 3.46	94.02 ± 3.23

It is trivial from the results that Canny edge as a condition for cGAN has outstanding performance, nearly perfect on more than one measure (Precision, Sensitivity, and F1). In the sigma (2 to 5) values examined, no consistent trend can be observed relating sigma values to classification accuracy. There is virtually no difference between the sigma_2 and sigma_5 evaluation metrics. This may indicate that this range of sigma values (i.e. this amount of edges) has no significant effect on the accuracy of the model. However, below 2 may pick up spurious edges from noise, and above 5 may result in too few edges leading to poor reconstruction.

We also evaluated the proposed method on an independent external dataset of 91 whole slide images. 270 high-power fields were sampled from the tumor bulk, outside the tumor bulk, and along the tumor front. Table 3 shows precision, sensitivity, and F1 scores for these 270 high power fields. Here, classification results reflect patches sampled from the HPFs that were either entirely tumor or non-tumor. These models were trained on the entirety of the original 24 slide training/validation dataset. A pathologist examined each HPF and identified the tumor border if it existed. 50 tumor and non-tumor samples were drawn from the ground truth of each HPF to compare pathologist performance alongside the proposed method. Results are reported in Table 3.

**Table 3:** Testing precision, sensitivity, and F1 score across 270 high power fields, sampled from an independent set of 91 slides from in the tumor bulk, outside the tumor bulk, and along the tumor front.

Condition	Our Framework			Pathologist		
	Precision	Sensitivity	F1	Precision	Sensitivity	F1
sigma_2	98.90 ± 2.43 (100.00)	88.53 ± 5.39 (90.00)	93.31 ± 3.07 (93.33)	99.67	99.18	99.43
sigma_5	82.19 ± 5.30 (82.61)	97.57 ± 3.28 (100.00)	89.10 ± 3.40 (88.89)			

A model trained on the entire 24 slide dataset was used for this evaluation. Reported as mean ± standard deviation (median). A pathologist evaluated each HPF.

A pathologist also classified each HPF (not tiles) into tumor and non-tumor categories. This resulted in a precision of 100, sensitivity of 98.90, and F1 score of 99.45. Taking a majority vote based tiles, our method achieved 100 on all metrics.

Finally, to further explore our framework’s ability to tolerate class imbalance, the tumor class of each fold as well the full 24 slide dataset was resampled to include 1/10 of the number of tiles of the respective non-tumor dataset. The results of the 4-fold, imbalanced cross validation are reported in Supplementary Table S1. The results of on 182 high power fields extracted from inside and outside tumor bulk and from the external testing dataset are reported in Table 4.

**Table 4:** Testing precision, sensitivity, and F1 scores across 182 high power fields, two sampled from each 91 slides in the tumor bulk and outside the tumor bulk.

Condition	Our Framework			Inception v3		
	Precision	Sensitivity	F1	Precision	Sensitivity	F1
sigma_2	99.72 ± 0.78 (100.0)	94.40 ± 5.45 (96.30)	96.90 ± 3.00 (97.74)	87.16 ± 14.03 (93.33)	99.97 ± 0.17 (100.00)	93.77 ± 6.25 (96.55)
sigma_5	95.61 ± 7.10 (98.44)	98.81 ± 2.41 (100.00)	97.02 ± 4.14 (98.53)			

Models were trained in previous manner but with extreme class imbalances – 10 non-tumor to 1 tumor tile. Reported as mean ± standard deviation and (median).

## Discussion and Conclusions

One of the advantages to the proposed model is its modularity. Most machine learning paradigms, Inception included, have a fixed number of class labels. Every time another class label or additional data are added, the model must be retrained with the updated dataset. In the case of neural networks, pre-trained models have been a major improvement on this issue. The large variety of images and resulting filters learned by these models allow us to train our own models in minimal time with either fine-tuning an existing model or by transfer learning. The modularity of the model proposed in this study could change this paradigm – if one wants to add another class to the existing data, simply train a cGAN on that data then add the generator of that cGAN to the collection of those trained on other classes. For example, our model trains two cGANs – one on tumor regions and one on non-tumor regions. If we instead want to detect cauterized tissue regions, we simply train a cauterized tissue model independently while leaving the other models untouched. Theoretically, the model would better reconstruct cauterized regions than the non-tumor model and tumor model.

Furthermore, unlike most deep learning models, our framework requires relatively small number of samples for training. We successfully trained our tumor model with fewer than 3,000 tiles. This enabled us to train our network from scratch within a few hours. This is in contrast to the number of trainable parameters in Inception (23,885,392) and the proposed method (89,860,228). As a side effect of this characteristic and modularity, problems such as class imbalance become less important – each individual network is learning a single class, not multiple. Finally, contrary to other deep learning models in histopathology, the method relies solely on edge information, making it theoretically less susceptible to variations (apart from focusing issues) in staining. We fully intend on exploring the extent of the modularity of this framework as well as the potential advantages discussed here in follow-up studies.

One of the underlying assumptions of our choice of tile size and magnification was that the best results with Inception v3 would also produce the best results for cGANs. This is not necessarily the case. There may be another, more optimal tile size and magnification for cGANs, and we could have tried to optimize the cGANs using similar experimental setup. Generally speaking, there is an optimal tile size and magnification for deep learning models, and models fail at certain combinations of size and magnification. However, our existing framework for Inception^7^ coupled with its shorter training time and smaller resource requirement compelled us to use 256 × 256 at 40x. An alternative viewpoint would be that we wanted our cGAN models to compete Inception’s best configuration.

The results in Table 2 for the proposed cGAN models trained on 256 × 256 pixel size tiles at 40x are promising. Under the Canny edge conditioning parameters, our model performed well with precision, sensitivity, and F1 values all above 93%. In earlier experiments with random dropping of data (results not reported), a large percentage of tumor tiles were misclassified as non-tumor in the validation set for Set 4. Briefly, we determined that there was a single, aberrant slide whose color varied greatly from that of the set 4 training set. Moreover, the number of tiles coming from this slide dominated the validation set in Set 4, as the number and amount of annotations for this slide happened to be larger than most. This suggests that Canny edge conditioning is less sensitive to stain variation.

Though classification results from an external test set in Tables 3 and 4 reflect slight wider distributed accuracies, they nonetheless indicate generalization of the proposed method. In our evaluation, 50 windows of 256 × 256 pixels were randomly from each HPF. Each reconstructed tile is compared to the original tile using MSE to determine the classification. As both tumor and non-tumor cGANs only see ‘pure’ tumor and non-tumor examples, they learn to reconstruct only their respective classes and not the other. Though the results are promising, they do not meet pathologist performance. However, the proposed method does not seek to meet or exceed pathologist performance in identifying tumor and stroma regions – pathologists can clearly identify these areas in H&E as evidenced by our results. It is in our next step – identifying tumor buds – in which we expect automated analysis will outperform pathologists. This proposed method seeks only to identify the tumor bulk (and thus invasive front) for a restricted region to identify tumor buds. Going forward, Table 4 results from a dataset with considerable class imbalance, and yet our method clearly outperforms transfer learning on Inception v3. For these reasons, we believe that method generalizes and effectively manages class imbalance.

**Figure 1:**
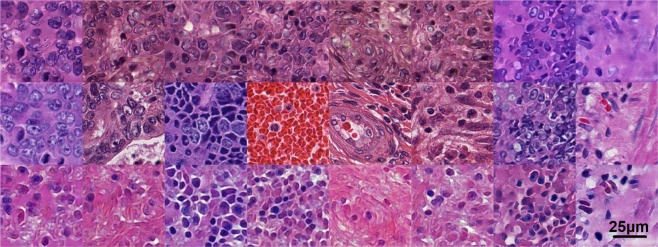
Sample tiles (middle row) and respective reconstructions using the tumor cGAN (top row) and non-tumor cGAN (bottom row), both conditioned with Canny (sigma = 2). First 6 columns from the left resulted in classification failures using MSE, and right 2 columns resulted in correct classifications. Sampled from set1 validation set.

Apart from MSE, different similarity and distance measures for comparison such as peak-signal-to-noise ratio and SSIM^30^ were utilized. These similarity and distance measures generally produced similar classification results as MSE. For the ease of implementation and efficiency, MSE was preferred over other measures. Like any classification method, the method also resulted in some false negatives. Figure 1 shows some failures of MSE to classify as well as some successes.

The failure of MSE to correctly classify patches is not due to color differences in the training and validation sets. In fact, the point of using edge information is to drain all color variation out of the images, preserving only structure. As a result of this conditioning, the network learns to map specific structure (edges) to specific features, like nuclei, cytoplasm, and cell type. The proposed method might fail if Canny fails to adequately detect edges. This could be caused by overexposure of the slide, too much stain, or too little contrast between stains.

**Figure 2:**
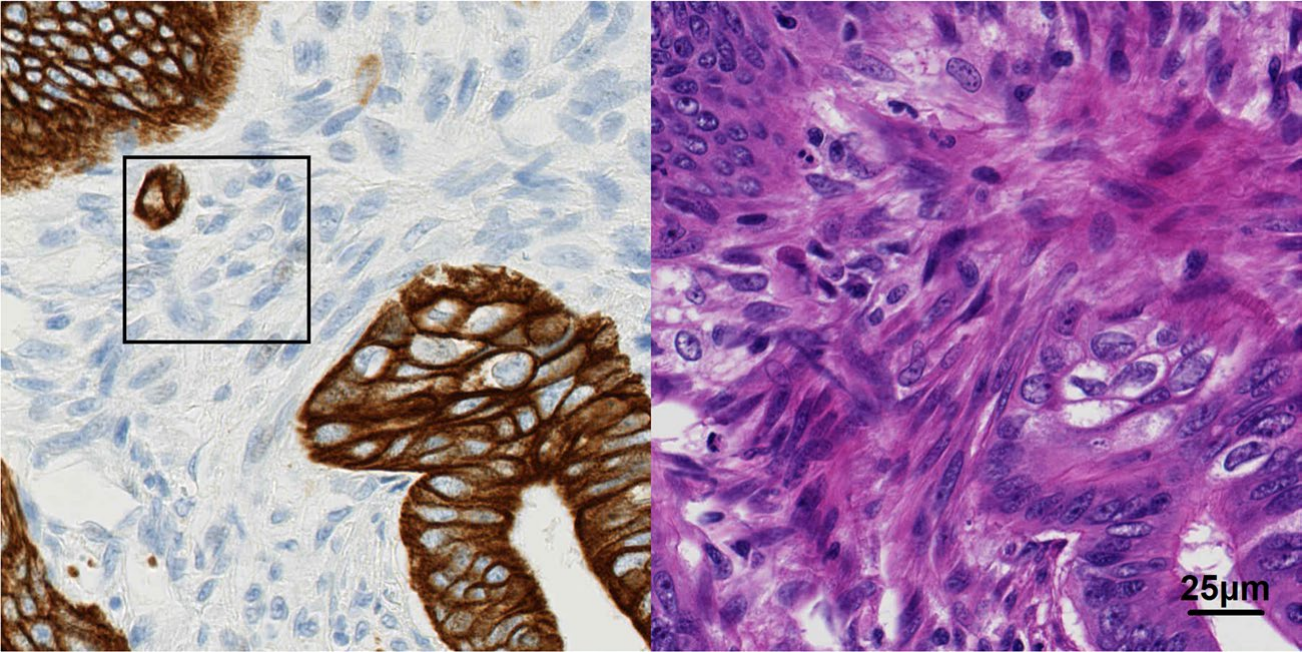
Issues with inter-tumoral tumor budding due to crude ‘windowing’ of the proposed method. Since the window is majority non-tumor, the patch is classified as non-tumor. Yet, there is a tumor bud.

The ultimate purpose of the proposed framework is to segment tumor bulk from the slide. This provides an estimated tumor invasive front, from which further analysis can focus on a small region around this boundary with the intention to identify tumor budding. However, the proposed method is not without drawbacks. Both cGANs are trained solely on ‘pure’ tumor and non-tumor tiles – neither model is trained on tiles at the tumor border. This may affect subsequent segmentation at the tumor border, which may limit detection of one (of two) kinds of tumor buds. Intra-tumoral tumor buds (i.e. those inside the bulk tumor) may fall under the radar, as they may be segmented along with the bulk tumor. This depends on the size of the window. At the moment, this window is 256 × 256 pixels. If this window fits into a non-tumor region within the tumor bulk, then there should be an issue. However, if this window overlaps too much with the tumor region, it may all be counted as tumor bulk, thus missing any potential tumor buds in that window. Figure 2 depicts these kinds of tumor buds and illustrates the potential problem described here.

On the other hand, identification of peri-tumoral tumor buds (i.e. beyond the tumor invasive front) does not require precise delineation of the tumor region borders. If the method results in an error around the tumor invasive front, it will presumably propagate to not more than 256 × 256 pixels.

Every model has its shortcoming and unfortunately the modularity of our method has these drawbacks. In addition, our method currently lacks the ability to quantify confidence in class predictions. Discriminative models often output a probability vector in which the value of each member reflects the probability of belongingness of that class. Our method simply classifies based on minimum MSE. We also recognize an additional problem – that our framework requires one model per class rather than one model over all. This is more computationally expensive, but this comes with the benefit of not having to retrain the model if considering and additional class.

In future, we will investigate the effect of color normalization, extent of modularity, precision of tumor front outline, and a fast, fully-convolutional method for our proposed model. This revised model will be trained on a larger dataset and be able to identify tumor buds^31^ from beyond the tumor front. Identification of tumor and non-tumor regions is a critical first step in identifying tumor budding, as tumor buds generally appear just beyond the tumor invasive front. We will also investigate the correlation of number of tumor buds to patient outcomes during our future study. Successful identification of tumor and non-tumor regions begets an easy delineation of the invasive tumor front. In turn, this provides a restricted region in which to detect tumor budding. Once potential tumor buds are detected from this region, they will be subject to nuclei detection, after which those clusters that contain more than five tumor nuclei will be discarded. Eventually, we will associate this with various clinical and pathological outcomes to provide quantitative evidence into how tumor budding affects prognosis.

## Methods

### Database

The IRB (2018C0098) for this study was approved by the Institutional Review Board, The Ohio State University. The IRB waived informed consent, as the data was anonymized. Our initial database consists of 24 adjacent whole-slide images of H&E and pan-cytokeratin stained CRC tumor sections acquired from 24 different patients. All slides were anonymized and digitized at 40x magnification using a high-resolution scanner (Aperio ScanScope XT, Vista, California) at 0.061 microns per pixel squared. Each pan-cytokeratin image was annotated for tumor and non-tumor (normal mucosa, submucosa, muscularis propria, inflammation, and fibrosis) regions and verified by gastrointestinal pathologist. These annotations were then manually transferred over to adjacent H&E slides and verified by the pathologist. Each annotation within H&E was then sampled for 256 × 256 tiles at 40x magnification using stereology^32^ – a square grid of points is laid across an image, and squares strictly inside the annotation are extracted as tiles – shown in Supplementary Fig. S2. The tiles within tumor regions were labeled as tumor tiles while the tiles within non-tumor regions were labeled as non-tumor tiles. These tiles were used for training and validation of the proposed modular network. An independent set of 91 adjacent H&E and pan-cytokeratin slides acquired from 91 patients was utilized as an external testing set. The IRB, informed consent process, digitization, and annotation process were the same as in the 24 slide set.

### GANs and cGANs

Generative adversarial networks (GANs) are a class of unsupervised machine learning algorithms in which two neural networks are pitted against one another in a zero-sum (i.e. adversarial) game. These two networks are referred to as the *generator* (G) and the *discriminator* (D). A discriminator is a familiar concept – it learns to map features to class labels. For example, modern convolutional neural networks (CNNs) attempt to classify images into specific categories. Generators are the opposite – they map labels to features, generating images from categories. In GANs, the generator learns to map a latent space to the distribution of the given dataset, while the discriminator learns to differentiate between samples from the given dataset and fraudulent samples produced by the generator. The common analogy is that of a counterfeiter and an expert. The generator learns to produce better counterfeits of the dataset as the discriminator learns to spot real and counterfeited data^33^. As both G and D are being trained, the weights in D are adjusted to *maximize* the probability of differentiating between real and fake images by the function

$$\log \,D(x)$$

where *x* is the ‘real’ probability. As it is a zero-sum game, the weights in G are adjusted to *minimize* the negation of this value

$$\log \,(1-D(G(z))$$

where *z* is random noise. These two equations constitute the cross-entropy loss function. Specifically, D and G participate in a two-player minimax game described by the function

$$\mathop{\min }\limits_{G}\,\mathop{\max }\limits_{D}\,{E}_{{\boldsymbol{x}} \sim {p}_{data}({\boldsymbol{x}})}[\,\log \,D\,({\boldsymbol{x}})]+{E}_{z \sim {p}_{{\boldsymbol{z}}}(z)}[1-\,\log \,D\,(G({\boldsymbol{z}})]$$

**Figure 3:**
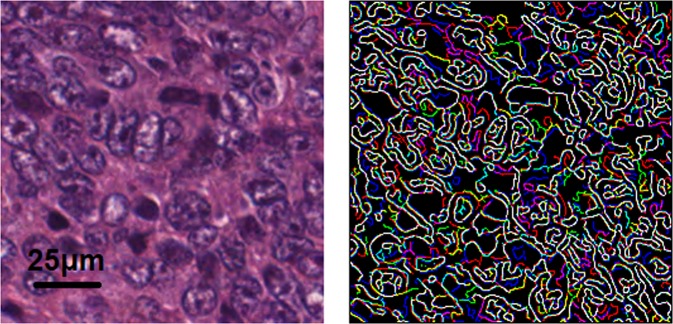
Manner in which the cGAN was conditioned. On the left is a *tumor tile*. The tile on the right shows the channel-wise Canny-detected edges. The red, green, and blue correspond to edges extracted from red, green, and blue channels, respectively.

This study utilizes cGANs. They are an extension of GANs in which input data for G and D are both *conditioned* on some additional information, in our case sample images (edges extracted from the 256 × 256 tiles with Canny edge detector) of H&E stains of CRC. This is carried out by feeding this extra information into both G and D in a joint representation. Thus, the minimax function becomes

$$\mathop{\min }\limits_{G}\,\mathop{\max }\limits_{D}\,{E}_{{\boldsymbol{x}} \sim {p}_{data}({\boldsymbol{x}})}[\,\log \,D\,({\boldsymbol{x}}|{\boldsymbol{y}})]+{E}_{z \sim {p}_{{\boldsymbol{z}}}(z)}[1-\,\log \,D\,(G({\boldsymbol{z}}|{\boldsymbol{y}})]$$

where *y* is the additional information (sampled images). Our method conditions the input data on *channel-wise Canny-detected edges*, as seen in Fig. 3. By only providing the edge (binary images) information, the network is forced to reconstruct the missing data. As a result, it learns the underlying distribution of the data through filters that accurately interpolate the missing values. It is necessary to mention that the *edge condition* is a 3-channel binary mask in which white pixels correspond to edges in an H&E image. The edge condition ensures that different anatomical structures are reconstructed at the same location as in the input image. This is crucial as it enables the pixel-to-pixel comparison (mean squared error) between the reconstructed and the original H&E tile. Structural similarity index measure (SSIM)^30^ was also tested for maintaining anatomical structures but was not utilized due to prohibitively long model training times.

Our cGAN implementation is based on *pix2pix*, a general-purpose cGAN solution to a variety of image-to-image problems^34,35^. It is capable of learning a mapping from an image input to an image output as well as a loss function during training^15^. The generator is implemented as a “U-Net” as described in^36^, and the discriminator is implemented as a standard convolutional neural network with softmax layer classification. Supplementary Fig. S3 shows an overview of the architecture. The generator’s loss function incorporated mean squared error and cross entropy. Supplementary Fig. S4 shows an overview of our framework.

### Inception v3

Inception v3 is a powerful deep convolutional neural network trained on the Imagenet^37^. For our purposes, it was used as a feature extractor where the auxiliary classifiers (i.e. the softmax layers) were retrained on our two class (tumor and non-tumor) dataset^7^. The learning rate was 0.01 with a mini-batch size of 100 over 3000 iterations.

### Training, validation, and testing sets

To reduce computational resources, a 4-fold cross-validation across all 24 training/validation slides was used to generate four distinct datasets. Each training ‘set’ seen in Supplementary Table S2 contains tiles (both tumor and non-tumor) from eighteen slides, and each validation ‘set’ contains the other six. Each set of six validation slides in these validation sets were independent from one another. Supplementary Table S2 details the number of tiles in each set. In general, the amount of tumor in CRC slides is relatively small compared to the amount of non-tumor, resulting in a relatively small amount of tumor tiles compared to non-tumor. As we are training separate cGANs for tumor and non-tumor tiles, it enables us to avoid the class imbalance problem that is usually encountered in binary classification. Finally, 270 high-power fields from an external testing set composed of 91 slides set were utilized for testing classification accuracies.

## Supplementary information

Supplementary Information

## Data Availability

Validation sets 1, 2, 3, and 4 from 24 H&E slides as well as external testing set of HPFs samples from 91 slides are available on Zenodo from 10.5281/zenodo.3377909.
