# Author Correction: A modular cGAN classification framework: Application to colorectal tumor detection

**DOI:** 10.1038/s41598-020-59307-6

**Published:** 2020-02-06

**Authors:** Thomas E. Tavolara, M. Khalid Khan Niazi, Vidya Arole, Wei Chen, Wendy Frankel, Metin N. Gurcan

**Affiliations:** 1 Center for Biomedical Informatics, Wake Forest School of Medicine, Winston-Salem, USA; 2 Department of Pathology, The Ohio State University, Columbus, USA

Correction to: *Scientific Reports* 10.1038/s41598-019-55257-w, published online 12 December 2019

This Article contains errors in the Reference list. References 7, 10, 13, 16, 18, 19, 20, 21, 25, 27, 28, 30, 33, 35, 36 and 37 were incorrectly given as:

7. Niazi, M. K. K. *et al*. In *Medical Imaging* 2018: *Digital Pathology*. 105810 H (International Society for Optics and Photonics) (2018).

10. Niazi, M. K. K. *et al*. In *Medical Imaging: Digital Pathology*. 86760I (International Society for Optics and Photonics) (2013).

13. Japkowicz, N. In *Proc. of the Int’l Conf. on Artificial Intelligence* (2000).

16. Qin, Z., Zhang, C., Wang, T. & Zhang, S. In *International Conference on Advanced Data Mining and Applications*. 1–11 (Springer) (2010).

18. Shaban, M. T., Baur, C., Navab, N. & Albarqouni, S. J. a. p. a. StainGAN: Stain Style Transfer for Digital Histological Images (2018).

19. Bayramoglu, N., Kaakinen, M., Eklund, L. & Heikkilä, J. In ICCV *Workshops*. 64–71 (2017).

20. Szegedy, C., Vanhoucke, V., Ioffe, S., Shlens, J. & Wojna, Z. In *Proceedings of the IEEE conference on computer vision and pattern recognition*. 2818–2826 (2016).

21. Liu, Y. *et al*. Detecting Cancer Metastases on Gigapixel Pathology Images (2017).

25. Kohl, M., Walz, C., Ludwig, F., Braunewell, S. & Baust, M. In *International Conference Image Analysis and Recognition*. 903–913 (Springer) (2018).

27. Tavolara, T. E. *et al. Colorectal tumor identification by transferring knowledge from pan-cytokeratin to H&E*. Vol. 10956 MI (SPIE, 2019).

28. Niazi, M. K. K. *et al*. *Generalization of tumor identification algorithms*. Vol. 10956 MI (SPIE, 2019).

30. Wang, Z. J. h. e. u. c. z. w. r. s. The SSIM index for image quality assessment (2003).

33. Goodfellow, I. *et al*. In *Advances in neural information processing systems*. 2672–2680 (2016).

35. Isola, P., Zhu, J., Zhou, T. & Efros, A. A. In 2017 *IEEE Conference on Computer Vision and Pattern Recognition (CVPR)*. 5967–5976 (2017).

36. Ronneberger, O. *et al*. U-Net Convolutional Networks for Biomedical Image Segmentation (2015).

37. Deng, J. *et al*. In *Computer Vision and Pattern Recognition. CVPR 2009. IEEE Conference on*. 248–255 (Ieee) (2009).

The correct references are listed below as refs. ^1–16^.
