# Supplementary material for: A modular cGAN classification framework: Application to colorectal tumor detection
Source: Sci Rep. 2019 Dec 12;9:18969. doi: 10.1038/s41598-019-55257-w (PMC6908583; doi:10.1038/s41598-019-55257-w)
Supplement: Supplementary file 1 — Supplementary Information [file 41598_2019_55257_MOESM1_ESM.docx]

A modular cGAN classification framework: Application to colorectal tumor detection

Thomas E. Tavolara, M. Khalid Khan Niazi, Vidya Arole, Wei Chen, Wendy Frankel, Metin N. Gurcan

Supplementary Figure S1. Comparison of various tile sizes and magnifications. 'train' is the average training accuracy across every hold-one-out training session (sampled every 10 iterations). 'train/MA5' is the moving average 'train' with a window of 5. 'val' is the average validation accuracy across every leave-one-out training session (also sampled every 10 iterations). 'val/MA5' is the moving average of 'val' with a window of 5. 'tr+vl' is the sum of 'train' and 'val' from steps 130-190. 'test' is the testing average across all hold-one-out models.


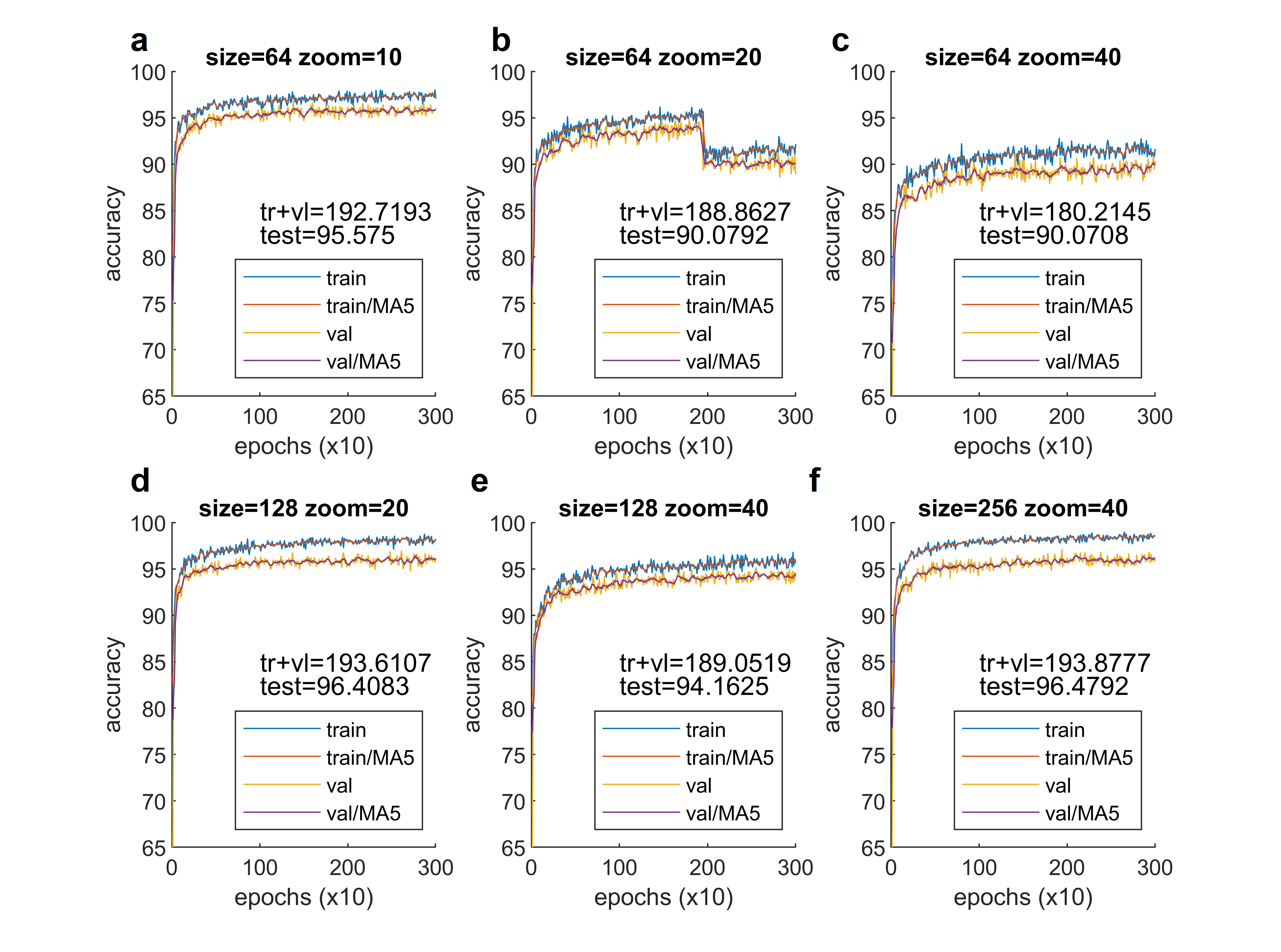


Supplementary Figure S2. Tile extraction from annotations. A grid is laid across the image, and each tile that falls completely within the boundary of the annotation is extracted as a part of the training or validation dataset.


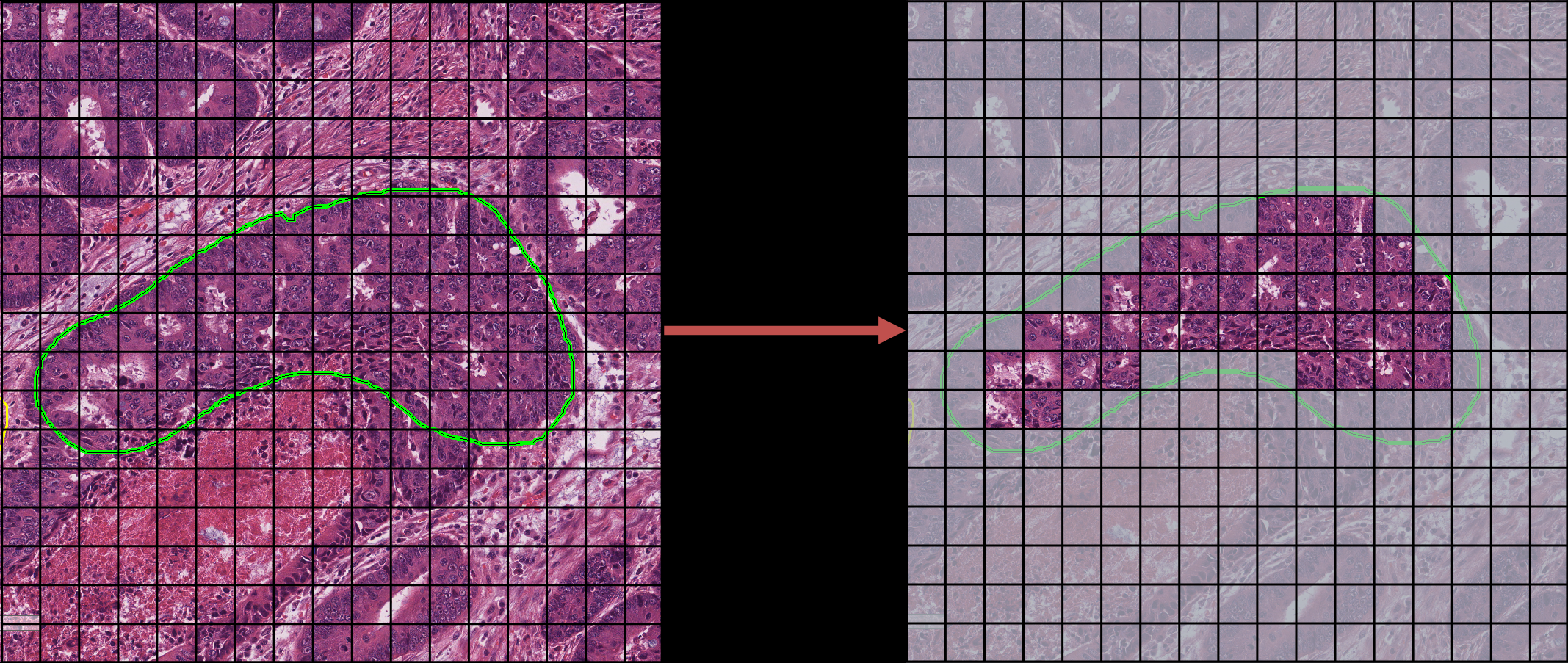


Supplementary Figure S3. An overview of a cGAN. The generator is trained on random noise (z) and conditioned on input data (y) to generate fake images (fake x). Eventually, these fake images look as real as real-world data (real x). The discriminator is trained to distinguish between the real images and fake images. The conditioned, real, and fake images shown here are directly drawn from one of our tumor cGANs.


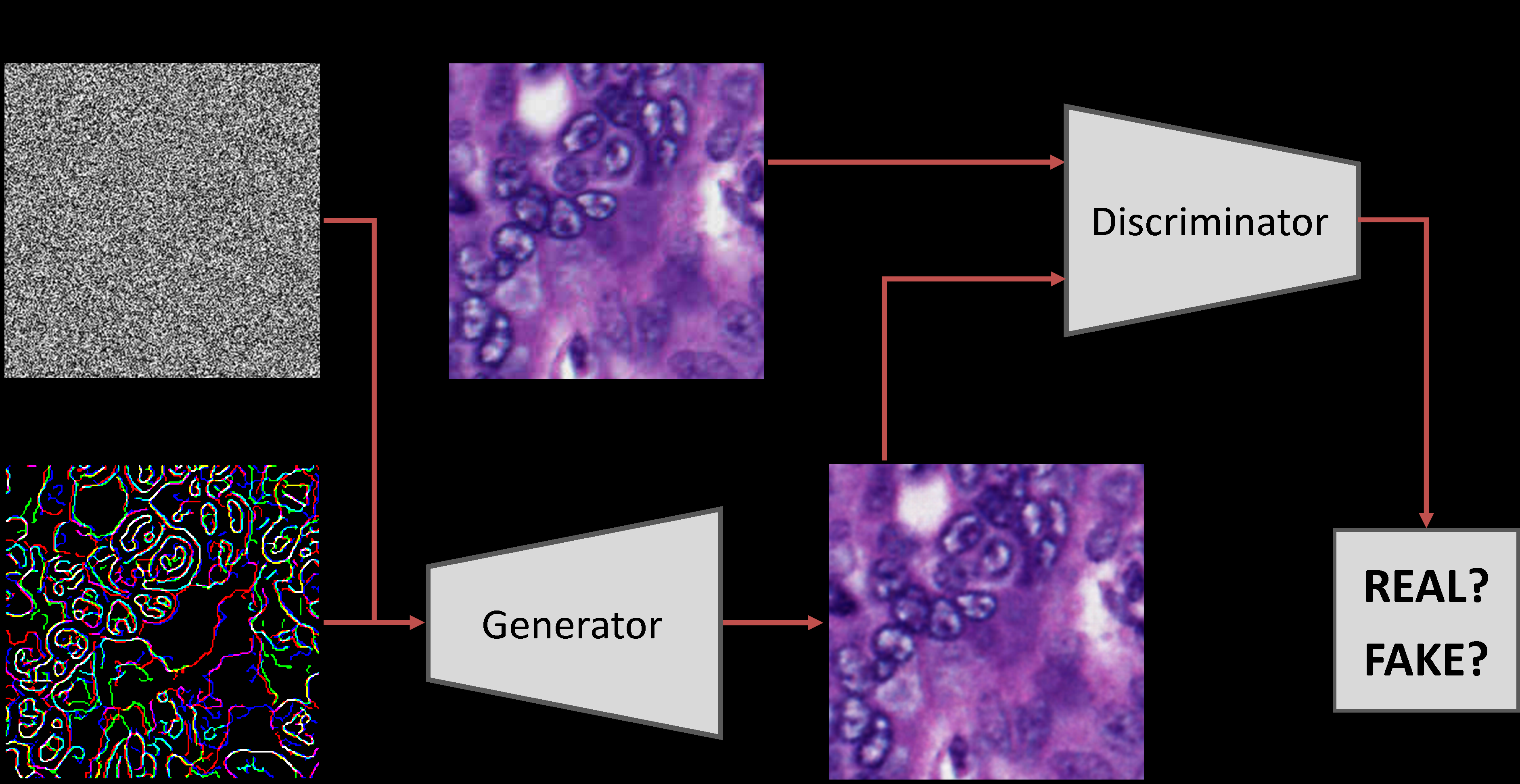


Supplementary Figure S4. An overview of the classification framework. The blue flowchart depicts how a ground truth is generated for the training of two cGANs. The red flowchart depicts how inference is done after training.


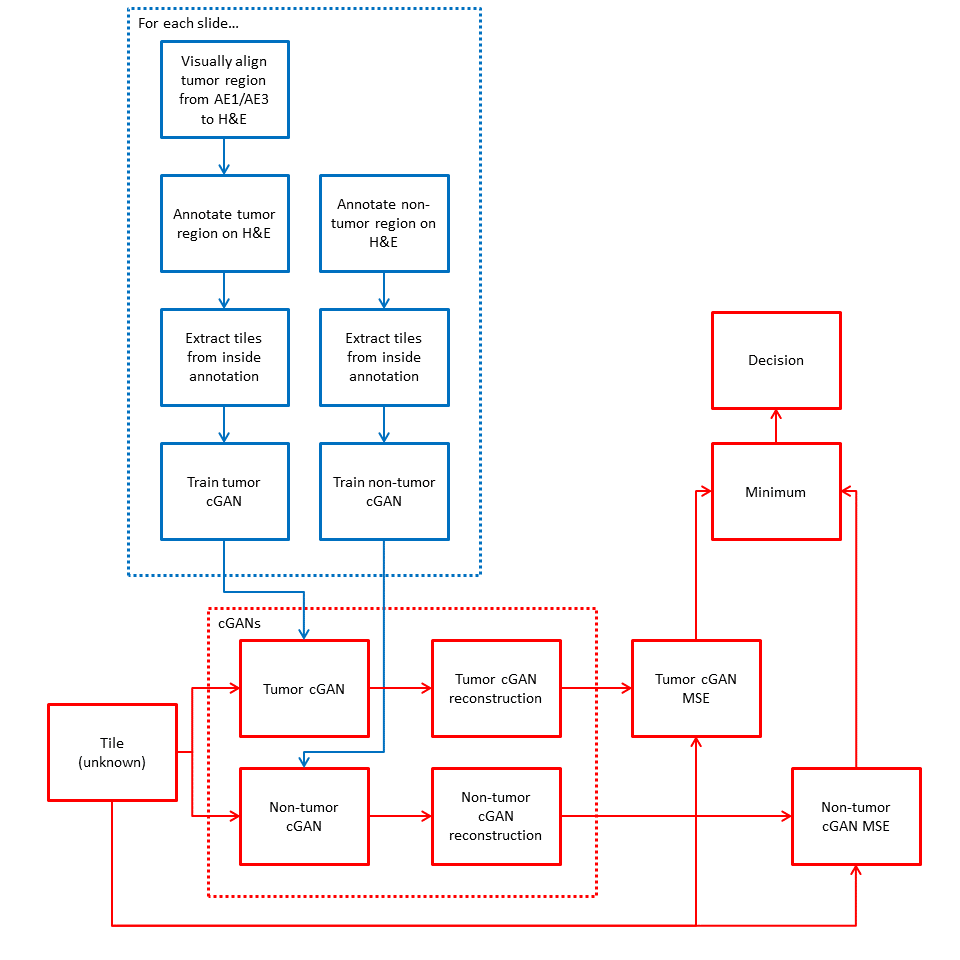


| Supplementary Table S1. Training and validation precision, sensitivity, and average F1 and standard deviations across each fold trained using cGANs with two Canny edge conditions. This is for the 4-fold, imbalanced cross-validation of 24 slides | | | | | | | |
| --- | --- | --- | --- | --- | --- | --- | --- |
| Model | Condition | Training | | | Validation | | |
|  |  | Precision | Sensitivity | F1 | Precision | Sensitivity | F1 |
| Our Model | sigma_2 | 99.24±.032 | 97.20±1.73 | 98.21±0.92 | 94.55±5.04 | 93.20±1.85 | 93.80±2.97 |
|  | sigma_5 | 99.30±0.51 | 97.55±1.13 | 98.40±0.39 | 95.13±4.44 | 93.05±3.46 | 94.02±3.23 |
| Inception | N/A | 98.90±0.22 | 98.52±0.13 | 98.71±0.09 | 96.25±2.68 | 97.92±0.81 | 97.10±1.32 |

| Supplementary Table S2. Dataset sizes. Generated from tumor and non-tumor annotations sampled for 256x256 tiles at 40x magnification. The relatively smaller number of tumor tiles in comparison to non-tumor tiles points towards the abundance of non-tumor regions in CRC slides. | | | | |
| --- | --- | --- | --- | --- |
|  | Training | | Validation | |
|  | Tumor | Non-tumor | Tumor | Non-tumor |
| set 1 | 8323 | 24582 | 3901 | 12490 |
| set 2 | 9388 | 28366 | 2836 | 8706 |
| set 3 | 10039 | 27862 | 2185 | 9210 |
| set 4 | 9746 | 32229 | 2478 | 4843 |
